# Simultaneously Low Rank and Group Sparse Decomposition for Rolling Bearing Fault Diagnosis

**DOI:** 10.3390/s20195541

**Published:** 2020-09-27

**Authors:** Kai Zheng, Yin Bai, Jingfeng Xiong, Feng Tan, Dewei Yang, Yi Zhang

**Affiliations:** 1 School of Advanced Manufacturing Engineering, Chongqing University of Posts and Telecommunications, Chongqing 430000, China; zhengkai2001@163.com (K.Z.); baiyin@cqupt.edu.cn (Y.B.); 2017214748@stu.cqupt.edu.cn (J.X.); tanfeng@cqupt.edu.cn (F.T.); zhangyi@cqupt.edu.cn (Y.Z.); 2 Department of Mechanical and Industrial Engineering, University of Toronto, Toronto, ON M5S 3G8, Canada

**Keywords:** simultaneously low rank and group sparse, Hankel matrix, singular value decomposition, periodic information index, bearing fault diagnosis

## Abstract

Singular value decomposition (SVD) methods have aroused wide concern to extract the periodic impulses for bearing fault diagnosis. The state-of-the-art SVD methods mainly focus on the low rank property of the Hankel matrix for the fault feature, which cannot achieve satisfied performance when the background noise is strong. Different to the existing low rank-based approaches, we proposed a simultaneously low rank and group sparse decomposition (SLRGSD) method for bearing fault diagnosis. The major contribution is that the simultaneously low rank and group sparse (SLRGS) property of the Hankel matrix for fault feature is first revealed to improve performance of the proposed method. Firstly, we exploit the SLRGS property of the Hankel matrix for the fault feature. On this basis, a regularization model is formulated to construct the new diagnostic framework. Furthermore, the incremental proximal algorithm is adopted to achieve a stationary solution. Finally, the effectiveness of the SLRGSD method for enhancing the fault feature are profoundly validated by the numerical analysis, the artificial bearing fault experiment and the wind turbine bearing fault experiment. Simulation and experimental results indicate that the SLRGSD method can obtain superior results of extracting the incipient fault feature in both performance and visual quality as compared with the state-of-the-art methods.

## 1. Introduction

Rolling element bearings (REB) are key components in the industrial mechanical systems such as wind turbine, high speed railway, aeroengine, etc., which are designed for long-term safe working -corrosion, some potential failures might occur on the rolling bearing surface [1]. Once the REBs are out of order, it will be not only highly expensive to replace them, but also seriously affect the operation reliability of mechanical systems [2,3]. To precisely keep abreast of the dynamic health condition for REBs, it is important to develop techniques to detect the incipient fault, in which vibration feature extraction is assumed to be an effective approach [4,5,6]. When the local fault happens in the REBs, there will be periodic impulses generated by striking the defect surface with rollers in the vibration signal [7,8]. However, the periodic impulses are expected for weak to be detected in the early fault stage, leading to a great challenge for bearing fault diagnosis [9,10].

One of the bottle-neck problems is to remove the substantial noise (namely, the Gaussian white noise and non-Gaussian noise) from the original vibration fault signal [11,12]. Over the past decades, many researches have been developed to fulfill this task for fault diagnosis. One line of research focuses on the bandpass filtering method, which seeks to accurately locate the resonant frequency band of REBs by designing a bandpass filter. The spectral kurtosis [13,14], the fast Kurtogram [15] and their improvements [16] are representatives among them. Another attempt is to represent the fault feature pattern with some well-selected basis. The typical techniques including the wavelet transform [17,18], the empirical wavelet transform [19,20] and dictionary construction [21]. Provided that the prior transform basis is seldom considered, several adaptive decomposition methods are extensively developed for fault feature extraction. The classical methods are the ensemble empirical-mode decomposition (EEMD) [22,23,24], local mean decomposition (LMD) [25,26], variational mode decomposition (VMD) [27,28] and singular value decomposition (SVD) [29,30]. The fundamental ideal of these methods is to decompose the original vibration signal into several components. Naturally, some fault-related components are selected to construct the fault feature. Among above methods, SVD is proved to have excellent ability to reveal the weak intrinsic pattern while suppress the noise with different distributions [31]. Owing to these merits, it has attracted considerable attention in recent years to estimate the incipient fault feature of the rolling bearings. 

A preliminary endeavor of SVD based feature extraction method is to establish the low rank domain for the Hankel matrix of the original signal through SVD operation. Therefore, singular components (SCs) with different characteristics are reliably projected into different lower-rank spaces [32]. This premise has recently guided research into the innovative and meaningful ideas of designing several effective approaches for fault feature extraction, which can be roughly divided into three categories. The first variety is the high-energy SCs oriented SVD method. For instance, Zhao et al. [33] proposed an SVD based fault diagnosis method for gear in the headstock. A difference spectrum that describe the sudden change status of singular values was proposed. By detecting the peak in the difference spectrum, the effective singular values can be selected. Qiao et al. [34] presented an SVD principle analysis and correlation coefficient-based method for bearing fault diagnosis. The index ρ  corresponding to the weak fault information was utilized to determine the effective SCs. It is hence the weak impulse signals can be detected. He et al. [35] put forward a time-varying singular value decomposition (TSVD) method for bearing fault diagnosis. TSVD adopted the row in the time-varying singular value matrix (TSVM) to identify the intrinsic structure of the raw signal. Xu et al. [36] proposed a slip Hankel matrix series construction and maximum singular value energy analysis method for fault diagnosis. The maximum singular value energy was proposed to separate the Oscillation dominated part and the noise dominated part. The above methods extracted high-energy SCs but may ignore the weak-energy ones. Nevertheless, the weak-energy ones may comprise the informative fault-related feature. In an attempt to yield better fault feature identification results, kurtosis-based SCs selection criterion for periodic fault feature extraction approach has been proposed in recent years. Golafshan R et al. [37] presented an SVD and Hankel matrix based de-noising method for bearing fault detection. More specifically, the kurtosis was employed to identify the periodic impulsive feature. Followed by this idea, Li and Liu et al. [38] presented a combined approach that integrate the singular value decomposition, singular value kurtosis and optimized frequency band entropy, named as SVD-SVK-OFBE, for bearing fault diagnosis. In this context, the Hankel matrix was reconstructed from the original vibration signal to performance the SVD, where the relative change rate of singular value kurtosis was adopted to determine the reconstructed order of singular values. Similarly, Li et al. [39] proposed a combined SVD-KSES technique, where the kurtosis of squared envelope spectrum (KSES) was employed to select the optimal SCs obtained by SVD. However, it is worth pointing that the kurtosis is a less ideal indicator depicting the fault feature information with the interference of large random impulses. In recent years, the informative SCs selection method has aroused attention increasingly as it can obtain the more accurate reconstructed fault feature signal. Zheng et al. [40] proposed an autocorrelation function periodic impulse harmonic to noise ratio (ACFHNR) index based on the SVD-Teager energy operator (TEO) for bearing fault diagnosis. The ACFHNR indicator was employed to measure the abundance of the periodic impulse fault feature of the SCs. Zhao and Jia et al. [41] proposed a reweighted SVD (RSVD) algorithm for rotating machinery fault diagnosis. They profoundly studied the periodic amplitude modulation (PAM) phenomenon of the fault feature. In light of this, an indicator called periodic modulation intensity (PMI) was introduced to estimate the informative level. Consequently, the informative SCs with PMI more than one could be highlighted. However, it should be noted that the PMI may be easily interfered by the harmonic component. Chen et al. [42] revamped the RSVD method. In the improved version, the SVD was applied to decompose the residual signal rather than the raw vibration signal. Moreover, the SCs were determined such that the PMI of the reconstructed signal is maximized.

According to above discussion, we can conclude that there are several major shortcomings in the previous studies, which can be remarked as follows: Firstly, the state-of-the-art methods mainly focus on the low rank property for the Hankel matrix of the fault feature pattern. Thus, the structure property is not sufficiently exploited, which cannot provide better estimation result of the periodic impulse fault feature. Secondly, the fault feature may be undermined in the low rank space. This may be attributed that the indicators such as kurtosis and PMI are not ideal enough to discriminate the fault feature pattern with heavy background noise. Thus, the fault feature in the low rank subspace is not the optimal. To address the above issues, a novel SLRGSD method for bearing fault diagnosis is proposed. Different from the current low-rank oriented fault diagnosis approach, the new diagnostic framework is proposed to prompt SLRGS property for the Hankel matrix of the fault feature pattern. Specifically, the periodic impulse fault feature is heightened by the periodic information (PI) indicator in the low rank space. Thus, the fault feature of the rolling bearing extracted by SLRGSD can be enhanced as compared with the current low-rank oriented fault diagnosis approaches. We summarize the main insights and contributions of this work as follows:

(1) We explore the intrinsic structures for the Hankel matrix of the fault feature pattern, indicating that it has the SLRGS property. To the best of our knowledge, it is the first time that the Hankel matrix of the vibration fault signal is modeled as a simultaneously low rank and group sparse matrix estimation problem.
(2) A novel SLRGSD framework is proposed for bearing fault diagnosis. The new framework is formulated to promote the SLRGS property. Moreover, the periodic impulses and interference components are split based on the PI index in the low rank domain. The PI is proved to have better fault feature discrimination ability than the PMI.
(3) The essential of the SLRGS property for improving the performance of SLRGSD method is verified by the numerical analysis and bearing fault diagnosis experiment. The results indicate that the proposed SLRGSD method can effectively enhance the fault feature.

The outline of this paper is as follows. In Section 2, the current SVD methods for bearing fault diagnosis are briefly introduced. In Section 3, the principles of the proposed SLRGSD method are detailed elaborated. Section 4 validates the effectiveness of the proposed method through numerical analysis. Besides, comparisons with the state-of-the-art methods are displayed. In Section 5, the effectiveness of the SLRGSD approach is verified by two experiments. Finally, we drawn the conclusions in Section 6. 

## 2. Review of Current SVD Methods for Bearing Fault Diagnosis

In general, the rolling bearings usually work in the harsh environment. A typical observed signal is constituted by the quasi-periodic transients generated from the inner race, outer race and ball fault of bearing, the interference harmonics and the background noise, which can be expressed as:

(1) $$y\left(n\right)=y^{i}\left(n\right)+y^{s}\left(n\right)+y^{n}\left(n\right),$$

where $y^{i}\left(n\right)\epsilon \;{\mathbb{R}}^{n\times 1}$ denotes the fault feature to be identified, $y^{s}\left(n\right)\epsilon \;{\mathbb{R}}^{n\times 1}$ is the interference harmonics while $y^{n}\left(n\right)\epsilon \;{\mathbb{R}}^{n\times 1}$ denotes the Gaussian white noise. Current Hankel based SVD methods aim to extract the fault features from interference harmonics and the noise in the low rank domain. In this section, we briefly introduce the primary process of the current Hankel based SVD methods for bearing fault diagnosis. Overall, the processes can be summarized as follows:

Step 1: Construction of the Hankel matrix

The collected signal is expressed as a time series, which cannot be directly processed by the SVD operation. In consequence, the 1-D collected time sequence is supposed to reshape into a 2-D matrix first for SVD processing. The Toeplitz matrix, cycle matrix and Hankel matrix and were proposed to fulfill this task. Because of the wavelet-like characteristics and zero phase shift property, the Hankel matrix is broadly applied [43]. In the following research, we will introduce the concept of delay embedding, which provides a more strict and general way to construct the Hankel matrix. Moreover, its inverse transform can be used to accurate change the 2-D estimated matrix into a recovered 1-D time sequences.

Step 2: Hankel matrix decomposition

For the time sequence y(n), we can obtain its constructed Hankel matrix Y. Then the Hankel matrix Y can be decomposed into the m sub-matrix through SVD operation in the low rank space, which satisfies:

(2) $$Y=U\Sigma V^{T\;}=\sigma_{1}u_{1}v_{1}^{\tau }+\sigma_{2}u_{2}v_{2}^{\tau }+\cdots \sigma_{m}u_{m}v_{m}^{\tau },$$

where $u_{i}$ is the columns vector of left singular matrix $U\in {\mathbb{R}}^{m\times m}$, $\sigma_{i}$ is singular value of the diagonal matrix $\Sigma \in {\mathbb{R}}^{m\times m}$ and $v_{i}^{\tau }$ is the column vector of the right singular matrix $V^{T\;}\in {\mathbb{R}}^{m\times l}$. Note that the singular values satisfy $\sigma_{1}\geq \sigma_{2}\geq \cdots \geq \sigma_{m}$.

Step 3: Reconstruction for feature extraction

After SVD operation, the signals with different characteristics can be projected into different spaces. For current Hankel based SVD method, the core strategy is to split the fault feature related SCs from interference harmonics and the noise related SCs in the low rank domain. The most energetic SCs preservation method [33,34], the maximum kurtosis SCs determination method [37], the singular value kurtosis SCs selection strategy [38] and the informative SCs selection approaches [40,41,42] for feature extraction are proposed in recent years. 

Based on above observation, we can find that the current Hankel based SVD methods are designed based on the low rank property of the fault feature. However, it should be noted that the intrinsic structure property for the Hankel matrix of the fault feature is not sufficiently exploited. Moreover, the kurtosis and PMI are not ideal enough to discriminate the fault feature with heavy background noise, which will undermine the fault feature in the low rank space. To obtain better estimation result, it is essential to explore a new framework for bearing fault diagnosis. 

## 3. Proposed SLRGSD Framework for Bearing Fault Diagnosis

**Figure 1:**
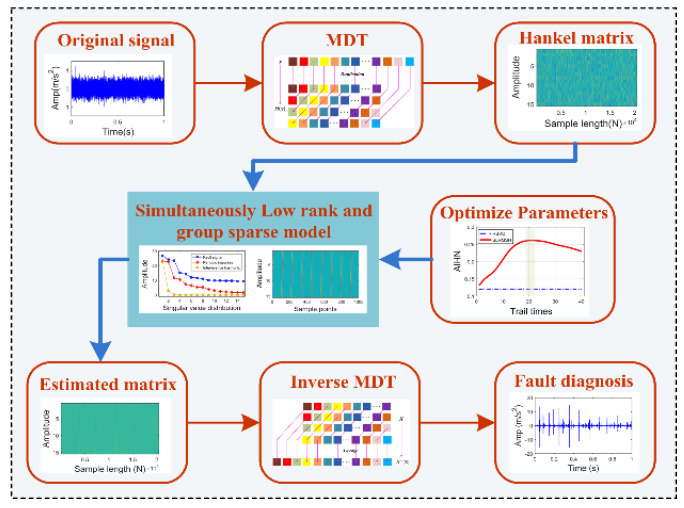
The framework of the proposed SLRGSD approach for bearing fault diagnosis.

The highlight of the SLRGSD approach is to establish a new fault diagnosis framework based on the SLRGS property for the Hankel matrix of the fault feature. The high-level steps of the proposed framework are described in Figure 1. Firstly, the original 1-D vibration fault sequence $x\in {\mathbb{R}}^{n\times 1}$ is transformed to the equivalent Hankel matrix $X\in {\mathbb{R}}^{M\times N}$ through delay-embedding transform (DET). After that, a simultaneously low rank and group sparse regularization denoising model is formulated through exploring the intrinsic Hankel matrix structure of the fault feature pattern. In this framework, a new weighted strategy is employed to highlight the fault feature in the low rank space while the sparse group lasso is utilized to promote the sparsity. Meanwhile, the regularization parameters of the proposed framework are optimized. Moreover, the incremental proximal algorithm is employed to achieve a stationary solution. Finally, the inverse delay-embedding transform (inverse DET) is implemented to transform the estimated matrix $\hat{X}\in {\mathbb{R}}^{M\times N}$ into the recovered 1-D signal $x_{rec}\in {\mathbb{R}}^{n\times 1}$.

### 3.1. DET and Inverse DET

The DET has aroused wide attention as a powerful tool for analyzing and recovering the time sequences, which reconstructs the attractor from the observed time-series signals [44]. The delay-embedding can be regards as a “Hankelization” process of time-series signal. Based on this point, the collected original bearing vibration signals $\left(x_{1},x_{2}\ldots x_{n}\right)$ can be transformed to a Hankel matrix $H_{\tau }\left(x\right)$ through delay-embedding, which is expressed as follows:

(3) $$H_{\tau }\left(x\right)=\left[\begin{array}{cccc} x_{1} & x_{2}\; & \cdots & x_{L-\tau +1}\\ x_{2} & x_{3} & \cdots & x_{L-\tau +2}\\ \vdots & \vdots \; & \ddots & \vdots \\ x_{\tau } & x_{\tau +1} & \cdots & x_{L} \end{array}\right],$$

where $H_{\tau }\left(x\right)$ is a duplicated matrix. Also, this process is regards as a standard DET. For a rigorous expression of the DET, we define $S\in {\left\{0,1\right\}}^{\tau \left(L-\tau +1\right)}$ as the duplication matrix, which satisfies [45]:

(4) $$vec\left(H_{\tau }\left(x\right)\right)=Sx,$$

Hence, the standard DET can be gained by:

(5) $$H_{\tau }\left(x\right)=fold_{\left(L,\tau \right)}\left(Sx\right),$$

where $fold_{\left(L,\tau \right)}$ is a folding operator from a vector to a matrix, which can be expressed as: $fold_{\left(L,\tau \right)}:{\mathbb{R}}^{\tau \left(L-\tau +1\right)}\to {\mathbb{R}}^{\tau \times \left(L-\tau +1\right)}$.

The forward delay embedding is consisted by the operation of duplication and folding. Thus, the inverse DET can be resolved into the corresponding inverse transforms, which includes a vectorization operation and the Moore-Penrose pseudo-inverse:

(6) $$S^{*}={\left(S^{T}S\right)}^{-1}S^{T},$$

Thus, the inverse DET for the matrix $H_{\tau }\left(x\right)$ can be obtained by:

(7) $${\bar{x}}_{rec}=H^{-1}{}_{\tau }\left(x\right)=S^{*}vec\left(H_{\tau }\left(x\right)\right),$$

**Figure 2:**
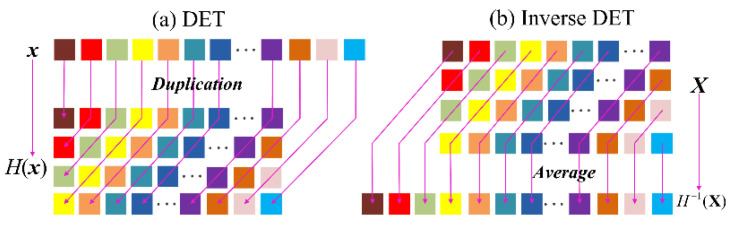
The schematic diagram of DET and the inverse DET.

The schematic diagram of DET and inverse DET is shown in Figure 2.

### 3.2. SLRGSD Model 

In the following contexts, we introduce how to establish the framework using the SLRGS property. For further analysis, the DET introduced in Section 3.1 is employed to project the 1-D signal $y\left(n\right)\epsilon \;{\mathbb{R}}^{n\times 1}$ into a 2-D Hankel space. Therefore, we can model the Equation (1) as:

(8) $$Y\left(N\right)=Y^{s}\left(N\right)+Y^{i}\left(N\right)+Y^{n}\left(N\right),$$

According to Equation (8), we can reveal the SLRGS property for the Hankel matrix of the periodic impulses fault feature, which are detailed elaborated as follows.

**Figure 3:**
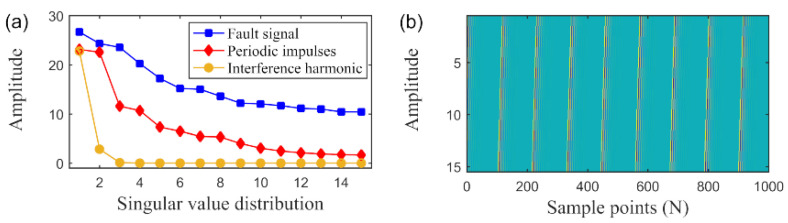
The low rank and group sparse property for the Hankel matrix of the periodic impulses fault feature. (**a**) The singular values distribution of the Hankel matrix of the different signals. (**b**) The colormap for the Hankel matrix of the fault feature.

- Low-Rank Property: The low rank property for the Hankel matrix of the bearing fault signal has been demonstrated by several studies such as Ref. [33,34,35,36,37,38,39,40,41,42]. We illustrate this property from Figure 3a, which provides an intuitive explanation about the low-rank property of the fault feature. Figure 3a demonstrates the singular values distribution of the Hankel matrix of the fault feature. It can be found that the signals with different characteristics can be projected into different spaces after SVD operation. In addition, the valuable SCs concentrate in a few large singular subspaces [41]. To this end, the Hankel matrix of the fault feature can be primarily estimated through the low rank nuclear norm constraint.
- Group sparse property: Another assumption believes that the periodic fault feature has the group sparse property, which were confirmed by the researches from Ref. [46,47,48]. On this basis, several denoising frameworks were designed for bearing fault feature detection. It is logical for us to assert that the Hankel matrix of the fault feature also owns the group sparse property if we make an analogy analysis. The colormap for the Hankel matrix of the fault feature is shown in Figure 3b. We can find that the Hankel matrix of the fault feature pattern exhibits an evident periodicity. There are periodic “bar code” in the colormap. While outside of the “bar code”, all elements can be regarded as zero. Therefore, it is reasonable for us to assume that the Hankel matrix of the fault feature has the group sparsity. This motivates us to pose a group sparsity cognizant estimator for the Hankel matrix, which will expect to realize a more accurate and robust estimation result.

Based on above analysis, it is not hard to conclude that the fault feature has the SLRGS property. Thus, we propose a new SLRGSD framework for bearing fault feature extraction. In this framework, we try to construct a new denoising model that in addition to well-fitting the fault feature signal with least square, it has low rank nuclear norm and the groups sparse for the Hankel matrix of the fault feature signal. To finish this task, we define the following optimization problem as follows:

(9) $$F\left(X\right)=\arg\underset{X}{\min}\left\{\frac{1}{2}∥Y-{X∥}_{F}^{2}+∥W⊙{X∥}_{*}+\left(1-\partial \right)\lambda \sum_{g=1}^{G}∥X_{g}∥{}_{2}+\lambda \partial {∥X∥}_{1}\right\},$$

where $\frac{1}{2}∥Y-{X∥}_{F}^{2}$ is quadratic data fidelity term, $∥W⊙{X∥}_{*}$ is the low rank constraint term, $\left(1-\partial \right)\lambda \sum_{g=1}^{G}∥X_{g}∥{}_{2}+\lambda \partial {∥X∥}_{1}$ denotes the groups sparse regularization function. Y is the Hankel matrix of the original vibration signal, X is the estimated Hankel matrix. ∂ and λ are the regularization parameters. In particular, λ controls the tradeoff between low rank and the group sparse regularization terms, ∂ balances the $l_{21}$ norm and $l_{1}$ norm of the sparse group term.

### 3.3. Proximal Gradient Descends for Solving SLRGSD

In this section, we exploit the solution of Equation (9) using the proximal gradient descend. According to Ref. [49], if the unconstrained convex optimization problem is differentiable, it can be solved by proximal gradient descends algorithm. The following minimization optimization problem can be depicted as follows:

(10) $$\underset{W\epsilon w}{\min}\sum_{i=1}^{m}f_{i}\left(W\right),$$

The $f_{i}\left(W\right)$ is the convex function. Based on Ref. [50], Equation (10) can be solved using the following procedure: Firstly, we derived the proximal operators of all the subfunction $f_{i}\left(W\right)$. After that, a computational sequence is established, where the proximal operator of $f_{i}\left(W\right)$ in the $\;i^{th}$ iteration can be obtained through the predecessor proximal operator of $f_{i-1}\left(W\right)$. Finally, the estimated result can be gained through repeating the procedure in a cyclic manner at each iteration. Note that F(X) is the sum of three convex functions, the incremental proximal algorithm proposed in Ref. [50] can be directly applied to our problem. The proximal operator of the function f(W) can be defined as:

(11) $$prox_{\lambda f\left(.\right)}\left(U\right)=arg\underset{W}{\min}\left(\frac{1}{2\rho }∥W-{U∥}_{F}^{2}+f\left(W\right)\right),$$

where ρ is the step size of the corresponding gradient step. 

Based on Equation (11), the derivation process for proximal operator of the $\frac{1}{2}∥Y-{X∥}_{F}^{2}$ can be transformed into a quadratic optimization problem. Thus, the proximal operator of the $\frac{1}{2}∥Y-{X∥}_{F}^{2}$ can be calculated through being the gradient of the objective function to zero. Therefore, the close-form solution of $\frac{1}{2}∥Y-{X∥}_{F}^{2}$ could be obtained as:

(12) $$prox_{\frac{1}{2}∥Y-{X∥}_{F}^{2}}\left(X\right)=\left(Y+\rho^{-1}X\right){\left(1+\rho^{-1}I_{N}\right)}^{-1},$$

For the proximal operator of the low rank term, it can also be gained by incremental proximal algorithm. Before further analysis, we first define soft thresholding operator of the matrix as follows:

(13) SHR(W)=sign(W)max(0,|W|−T),

where T is the threshold matrix, W is the diagonal matrix. The diagonal elements of it are assumed to shrink by thresholding parameters contained in a vector. The threshold function of the diagonal elements can be expressed as $S\mathrm{HR}\left(\omega_{ij}\right)=\mathrm{sign}\left(\omega_{ij}\right)\max\left(0,\left|\omega_{ij}\right|-\mu_{ij}\right)$, where $\;\mu_{ij}$ is the threshold parameter. Therefore, the proximal operator of the nuclear norm can be calculated via a soft-thresholding operation on the singular values [51], which can be expressed as follows:

(14) $$prox_{∥W⊙{X∥}_{*}}\left(X\right)=U\mathrm{SHR}\left(X\right)V^{T},$$

Note that another challenge should be resolved, that is, the valuable SCs should be determined through an appropriate rule. Concerning the periodic impulsive characteristic for the fault feature of the rolling bearing, we redefine an equivalent function of SHR(X), which can be expressed as follows:

(15) $${\mathrm{SHR}}_{W}\left(X\right)=\sum_{i=1}^{M}\omega_{i}\sigma_{i}\left(X\right),$$

where $\omega_{i}$ is the weight vector that represents the weight of the singular component $\sigma_{i}\left(X\right)$, M is the number of SCs. Hence, the valuable fault SCs could be selected based on $\omega_{i}$, where $\omega_{i}$ is defined as follows:

(16) $$\omega_{i}=\{\begin{array}{c} \;\;0,\;\;\;\;\;\;\;\;\;\;\;{\mathrm{PI}}_{i}<\max\left({\mathrm{PI}}_{i}\right)\;\\ 1,\;\;\;\;\;\;\;\;\;\;\;{\mathrm{PI}}_{i}=\max\left({\mathrm{PI}}_{i}\right) \end{array},$$

In Equation (16), the PI indicator is proposed to quantitatively measure the fault information in the vibration signal. The PI index [52] is synthesis by the ratio of autocorrelation impulses harmonic to noise (AIHN) and kurtosis indicator, which can be obtained as follows:

(17) PI=AIHN·kurtosis,

where kurtosis represents the impulsive information while AIHN reflects the periodic information of the rolling bearing fault signal. Therefore, the PI index can measure the periodic impulsive fault feature of the rolling bearing. It should be noted that if more feature information contained in the vibration signal, the larger PI index will be.

Moreover, for a clear expression, we define the groups sparse term as follows:

(18) $$SGL\left(X\right)=\left(1-\partial \right)\lambda \sum_{g=1}^{G}{∥X}_{g}∥{}_{2}+\lambda {∥X∥}_{1},$$

As indicated in Ref. [53,54], the proximal operator of SGL(X) has the following closed form solution:

(19) $$prox_{SGL}\left(X\right)=\mathrm{soft}\left(y,\partial \lambda \right){\left(\frac{∥\mathrm{soft}{\left(y,\partial \lambda \right)∥}_{2}-\left(1-\partial \right)\lambda }{∥\mathrm{soft}{\left(y,\partial \lambda \right)∥}_{2}}\right)}_{+},$$

where the soft function is defined as:

(20)  soft(α,β)=sign(α)max(|β|−α,0),

Combining the closed form solutions of Equation (12), (14) and (19), we can gain a satisfying stationary result using the proximal gradient descends for solving the SLRGSD approach. The detailed processes are summarized in Algorithm 1.
**Algorithm 1** Proximal gradient descends for solving SLRGSD.Require: Hankel matrix X, regularization parameter ∂ and λ, maximum iteration N,  ρ=1.
1. $k=1,\;N=30,A=1+\rho^{-1}I_{N}$.2. for k≤N do
3.
$X^{k}=\left(Y+\rho^{-1}X^{k-1}\right)A$.4. update $X^{k}$ through Equation (14).
5. update $X^{k}$ through Equation (19).6. until convergence
7. end for
8. Obtain the estimated matrix $X_{e}=X^{k}$.

### 3.4. Fault Diagnosis Procedure of Rolling Bearing Using SLRGSD

**Figure 4:**
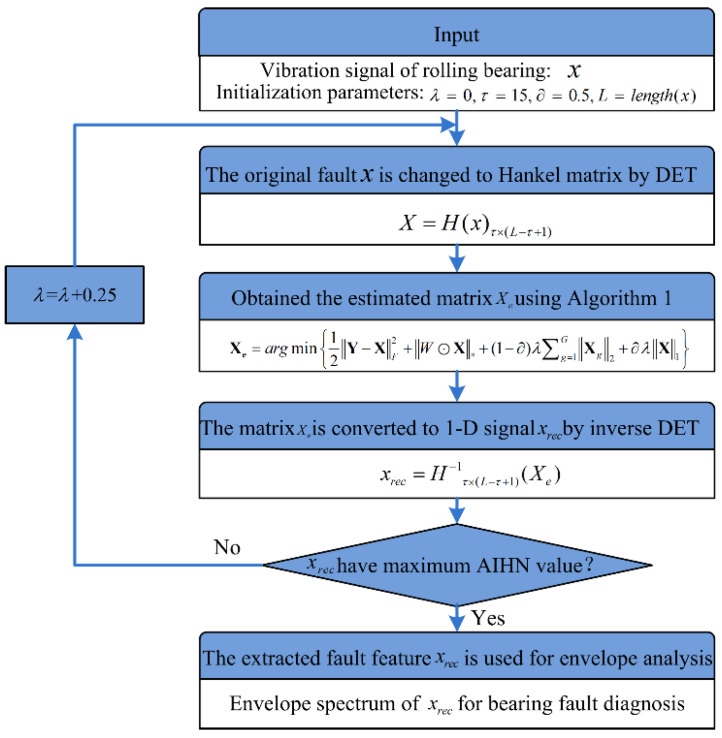
Fault diagnosis procedure of rolling bearing based on SLRGSD.

To extract the weak fault feature from the signal with heavy background noise, a fault diagnosis procedure for the rolling bearing based on SLRGSD is proposed. The detailed processes are as follows:

Firstly, the original vibration fault signal $x\in {\mathbb{R}}^{n\times 1}$ is transformed to Hankel matrix $X\in {\mathbb{R}}^{M\times N}$ through DET. After that, the proposed Algorithm 1 is employed to obtained the estimated matrix $X_{e}$. Thirdly, the inverse DET is implemented to convert the estimated matrix $X_{e}\in {\mathbb{R}}^{M\times N}$ into the recovered 1-D signal $x_{rec}\in {\mathbb{R}}^{n\times 1}$. It should be noted that the parameters of SLRGSD are configured as τ=15,∂=0.5 while λ can be optimized through finding the maximum value AIHN of the extracted fault feature. The parameters selection rule of SLRGSD will be detailed explained through numerical analysis in Section 4. Finally, the denoised signal $x_{rec}$ is employed to obtain the envelope spectrum for bearing fault diagnosis. The algorithm flowchart of using the proposed SLRGSD for bearing fault diagnosis is illustrated in Figure 4.

## 4. Numerical Analysis

In this section, the performance of the proposed SLRGSD approach is evaluated by the simulation signal with heavy background noise. Firstly, we introduce the simulation signals and the performance metrics. Then, the assessment of the PI index and the regularization parameters configuration strategy are conducted. Moreover, the role of the SLRGS property is investigated with the assistance of AIHN indicator. Finally, the effectiveness of the proposed SLRGRD method and its comparison with other methods are depicted in detail. 

### 4.1. Simulation Setup

In the numerical experiments, the observed simulation signals generated by the model mentioned in Section 3.2 are used to assess the performance of the SLRGSD approach. For this purpose, the rational evaluation of the feature recognition performance for SLRGSD method can be gained by designing the various combinations of interference harmonics and noise. Considering that the electrical interference or knocks on the test rig always exists in the real environment, thus the large random impulses are added in the tested signal. Specifically, the underling simulation signal can be expressed as follows:

(21) $$\begin{array}{cccccccc} x(t)= & \overset{K}{\underset{k=1}{\sum }}A\cdot s_{k}\left(t-kT-\tau \right) & + & \overset{M}{\underset{m=1}{\sum }}B_{m}\cdot sin\left(2\pi f_{m}t+\beta_{m}\right) & + & \overset{N}{\underset{n=1}{\sum }}C_{n}\cdot s_{n}\left(t-T_{n}\right) & + & n\left(t\right)\\ & ⏟ & & ⏟ & & ⏟ & & ⏟\\ & \text{Periodic impulses} & & \text{Interference harmonics} & & \text{Large random impulses} & & \text{Noise} \end{array},$$

The first term of x(t) is the periodic impulses, the second term denotes the interference harmonics, the third term is the large random impulses while the fourth term is the background noise. It is worth mentioning that s(t) represents the impulse response function, which can be modeled as:

(22) $$s\left(t\right)=e^{-\alpha t}\sin\left(2\pi \;f_{r}t\right),$$

**Figure 5:**
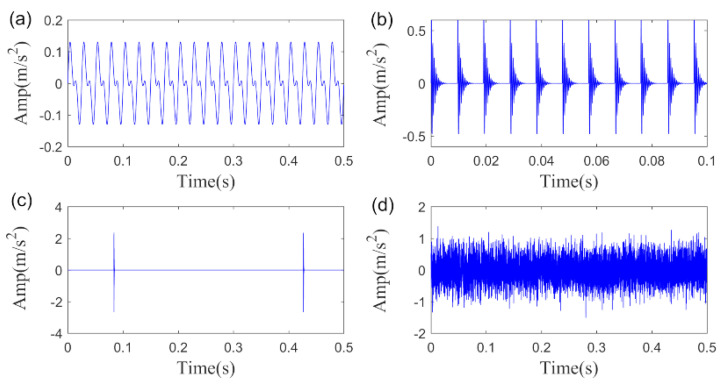
Simulation signals: (**a**) interference harmonics, (**b**) periodic impulses fault feature, (**c**) large random impulses, (**d**) the noise signal.

In summary, the parameters of Equations (21) and (22) are explained as follows: T is the cyclic fault periodic , A denotes the amplitude of periodic impulse sequences, τ is the random slippage,$\;B_{m}$ is the amplitude of the interfere harmonics, $f_{m}$ and $\beta_{m}$ are the rotating frequency and phase. $T_{n}$ and $C_{n}$ denote the occurred time and the amplitude of the large random impulses. α and $f_{r}$ represent the damping coefficient and the resonance frequency, respectively. The above parameters are set as: T=1/105, A=0.9, $\tau_{k}=0.70\%T$, $\;f_{r1}=2000\;\mathrm{Hz}\;$, $f_{r2}=6000\;\mathrm{Hz}$, $C_{1}=0.1,\;C_{2}=0.02,\;f_{m}=40\;\mathrm{Hz},\;\beta_{m}=0\;$. The sampling frequency $f_{s}\;$ is set as 12 kHz while the length of the simulation signal is set to 6000. The simulation signals are plotted in Figure 5.

We adopt two indicators, namely the approximate entropy (ApEn) [55] and the SNR output [56], to qualitatively assess the performance of the SLRGSD method. The ApEn can describe the repeatability and “regular” of the time sequence. When the time sequence has substantial fluctuation of irregularity, it will have larger ApEn value [56]. Therefore, it could be used to measure the noise level of the extracted fault feature in time domain. However, the ApEn may get a misleading result if the extracted fault feature is more “regular” while less “informative”. Therefore, the SNR output is an ideal complementary indicator to accurately reflect the information of the extracted fault feature in frequency domain. It can distinguish the target frequency from the interfering frequencies. Apparently, larger value of SNR output means that more target frequencies are detected in the fault feature [55].

### 4.2. The PI Index Assessment

**Figure 6:**
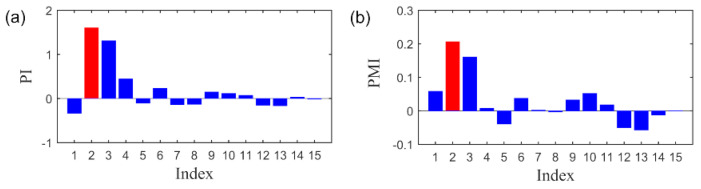
The indexes value of each SCs. (**a**) The values of PI index. (**b**) The values of PMI index.

Before subsequent analysis, we examine the advantages of PI index for constructing the weighted nuclear norm. The simulation signal with SNR = −13.2 dB generated in Section 4.1 is employed to illustrate this point. With SVD operation, the PMI and PI index corresponding to each SC can be computed. As indicated by the RSVD method [41], informative SCs were selected if PMI > 1. However, this criterion may lose effect when the incipient fault feature is excessively weak. As shown in Figure 6, the largest PMI value is only 0.18, which is far behind than 1, indicating that it is hard to locate the informative SCs with the threshold value setting as 1 if the fault feature is too weak. Therefore, the SC with maximum indicator value is selected as the feature component. Specific to this case, SC#2 is regard as informative fault feature since it has the largest PMI and PI value. Moreover, with visually inspection, we can observe that the amplitude of fault related SC#2 measured by PI is relatively larger than PMI, while the amplitudes related to the interference SCs are smaller. It is empirically demonstrated that the PI may have better ability than PMI to identify the fault feature. 

**Figure 7:**
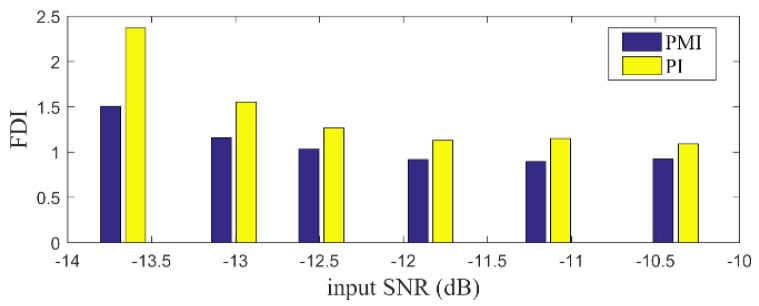
The FDI value of PI and PMI with different simulation signals.

We further define a new indicator to quantitatively evaluate the discrimination ability of fault feature information for the PMI and PI. Inspired by the definition of SNR, we defined feature discrimination indicator (FDI) as follows:

(23) $$\mathrm{FDI}=\frac{SC_{u}}{\sum_{i}SC_{i}-SC_{u}},$$

where $SC_{u}$ is the value of the indicator related to the fault feature SCs while $\sum_{i}SC_{i}-SC_{u}$ denotes the value of the interference SCs. It is evident that larger value of FDI implies better feature discrimination for the indicators. Utilizing the definition, we calculate the FDI of PMI and PI for the simulation signal mentioned above, which PMI equals to 1.2876 while PI is 1.8870. The result confirms the observation that the PI index indeed has better fault feature discrimination ability than PMI. Moreover, in order to comprehensively assess the performance of PMI and PI, we compute the FDI value with simulation signals under the different level of noise. The results are plotted in Figure 7. Obviously, we can find that the PI index is superior than PMI in detecting the weak fault feature. Thus, the PI index performances better to highlight the periodic fault feature in the low rank space. 

### 4.3. Regularization Parameters Configuration Strategy 

SLRGSD method contains three key parameters, including the delay time τ, the regularization parameters ∂ and λ. In this research, ∂ and λ are both restricted in the range of (0,1). For the delay time τ, it could be chosen about three times the number of components in the raw signal [41]. We assume that the fault vibration signal contains four parts, which are quasi-periodic impulses, the interference harmonics, the large random impulses and the Gaussian white noise. Therefore, the τ should satisfies τ>12. Without loss of generality, the delay time τ is fixed as τ=15. For the group sparse bound term, ∂ determines the balance of ${∥X∥}_{1}$ and $\sum_{g=1}^{G}∥X_{g}∥{}_{2}$. In other words, if ∂=0, the groups sparse term will change into the well know $l_{21}$ norm while if ∂=0, it will degenerate into the plain sparsity $l_{1}$ norm. Meanwhile, according to Ref. [55], the group sparsity of the solution is determined by the magnitude of the penalty parameter λ. Note that the regularization parameter of the low rank bound term is defaulted to be 1. Consequently, the penalty parameter λ is assumed to be a weighting factor that controls the low rank and group sparse term. More specifically, if λ=0, the cost function of Formula (9) only has the low rank term, thus, it will be similar with the traditional SVD based feature extraction solution. Otherwise, if λ=1, the cost function has the SLRGS property. However, it may over-fitting the Hankel matrix of the fault feature, which hinders the performance of SLRGSD method. 

**Figure 8:**
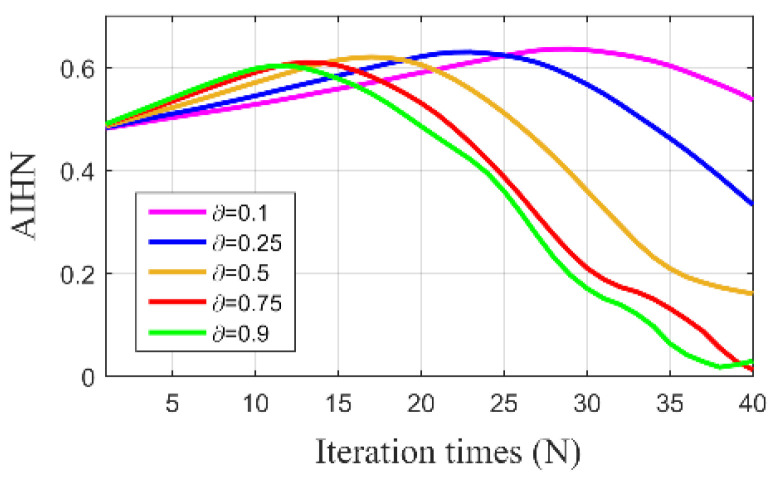
The trajectory of AIHN value with different parameters ∂.

We first analyze the influence of parameter  ∂ to the performance of the SLRGSD approach. Within this process, the SNR of the simulation fault signal is set as −12.8 dB. Five typical parameters ∂=0.1, 0.25,0.5, 0.75 and 0.9 are fixed while the parameters λ increases from 0 to 1 with an interval of 0.025. At this case, we observe the property of the extracted feature signal using the SLRGSD approach. To visually demonstrate the influence of parameter ∂, AIHN is adopted as the evaluation indicator for observation. The results are plotted in Figure 8. We can find that when λ is changing from 0 to 1, there are always maximum AIHN with different penalty parameter ∂. Interestingly, it seems that the maximum AIHN change little when ∂ is in the range of 0.1≤∂≤0.75. In order to balance the $l_{21}$ norm and $l_{1}$ norm, we fixed ∂=0.5 for the SLRGSD approach. 

Regarding the tuning parameter λ, it balances the property of low rank and group sparsity. Therefore, the performance of the proposed SLRGSD method is directly determined by the parameter λ. In other words, the SLRGSD method is robust to the parameter λ. This robustness is a basic phenomenon for the proposed sparse regularization problem, leading to the under-fitting to over-fitting to the Hankel matrix of the fault feature. As a result, it is reasonably for us to deploy the optimal regularization parameter λ via searching the unique maximum AIHN value. The detail of this process will be explained in the following subsections.

### 4.4. The Role of SLRGS Property 

The highlight of the SLRGSD method for enhancing the incipient fault feature is to prompt the SLRGS property for the Hankel matrix of the fault feature as compared with the current low-rank based methods. Note that the penalty parameter λ balances the property of low rank and group sparsity. Therefore, we exploit the influence of parameter λ to the performance of SLRGSD approach for elaborating the effect of SLRGS property. With this process, we explore the relationship between parameter λ and the AIHN value of the extracted feature signal, where the parameter λ is changing from 0 to 1. In a similar way, the parameter λ is increment from 0 to 1 with an interval of 0.025. Under this circumstance, we obtain the trajectory of AIHN with four group of simulation signal, where the SNR of them are set as −11.49 dB, −12.16 dB, −12.80 dB and −13.39 dB, respectively, as shown in Figure 8. It can be found that the trajectory of AIHN abides by a similar rule with different simulation signals. In the initial stage, the low-rank property plays a dominant role. When the parameter λ grows, the AIHN also gradually increases. After that, the AIHN value reaches the peak point and then shows a trend of gradually decreasing when the parameter λ is increasing. This is because the group sparse property become the primary role, which restrains the low rank property. Overall, it can be found that the SLRGS property plays a crucial role of attaining the maximum AIHN value. Additionally, we can conclude that the optimal feature will be obtained if the parameter λ is properly selected because of the SLRGS property. 

**Figure 9:**
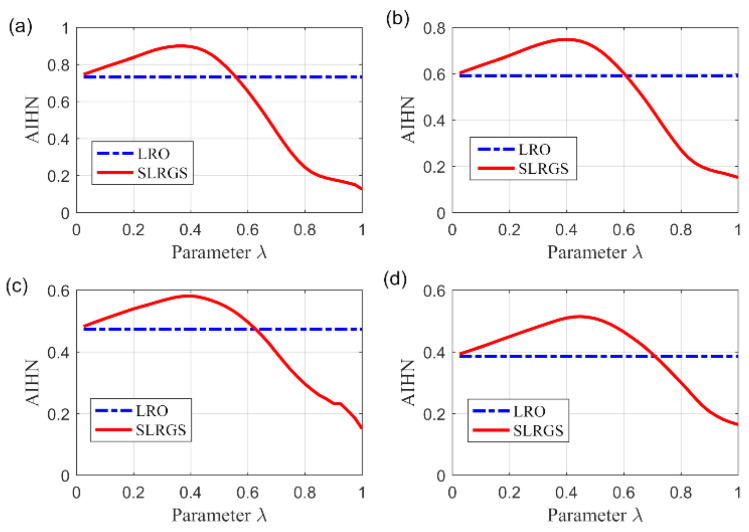
The relationship between the parameter λ and the AIHN value with different simulation signal. (**a**) Input SNR = −11.49 dB. (**b**) Input SNR = −12.16 dB. (**c**) Input SNR = −12.80 dB. (**d**) Input SNR = −13.39 dB.

Moreover, we compare the SLGRS property with the low rank-oriented (LRO) strategy for extracting the fault feature. For a fair comparison, the implementation processes of the LRO strategy are shown as follows: Firstly, the Hankel matrix of the original signal is decomposed into several SCs via SVD operation. After that, the PI values of all SCs are calculated for evaluation. Finally. the SC with maximum PI value is selected to reconstruct the fault feature signal. The AIHN values for the LRO strategy of the simulation signals with different noise level are also displayed in Figure 9. It is clearly that the maximum AIHN values of the extracted fault feature signal using SLRGSD method are larger than the LRO strategy if appropriate parameter λ is selected. For example, in Figure 9b, we can conclude that the optimized λ=0.4. Consequently, the AIHN = 0.7477 can be computed for the extracted fault feature using SLRGSD method. However, the AIHN of the extracted fault feature gained by LRO strategy is only 0.5911. On average, the optimal information fault feature acquired via SLGRSH is over 20% higher than the LRO strategy based on the simulation signals. 

In summary, we can draw the conclusions that the SLGRS property is a critical and indispensable strategy to enhance the weak fault feature for the proposed method. Therefore, it is reasonable to affirm that SLGRSH method provides a more robust and effective way of extracting the fault feature for bearing fault diagnosis.

### 4.5. SLRGSD Versus Other Methods

In this section, the fault simulation signal generated in Section 4.1 is employed to visually illustrate the effectiveness and superiority of the proposed SLRGSD method. For comparison, the RSVD [41] and the parameters optimized variational mode decomposition (PO-VMD) [57] methods are employed to undertake the same fault diagnosis task.

- RSVD: RSVD method decomposes the Hankel matrix of the original signal into several SCs via SVD operation. Then the information of periodic impulses in each SC is measured through the PMI indicator. When PMI > 1, the relevant SCs was selected to reconstruct the fault feature signal. Hence, RSVD method is developed based on the low rank property for Hankel matrix of fault feature signal. In this research, it should be noted that the reconstructed fault feature is determined by the SC with maximum PMI as the fault feature is contaminate by the heavy noise.

- PO-VMD: VMD method has attracted wide attention for extracting the bearing fault feature. The fundamental ideal is to decompose the raw input fault signals into a serial of sparse discrete modes. The sensitive sub-components can be affirmed based on tailored statistical indicators such as the kurtosis, the entropy, etc. The parameters such as the mode frequency bandwidth control parameter α and the number of modes K play a significant influence to the performance of the decomposition results. Like the method proposed in Ref. [57], the moth-flame optimization (MFO) algorithm is employed to optimize the key parameters of VMD to gain a better fault feature detection result. Meanwhile, we adopt the PI index to construct the fitness function. The sensitive sub-component can be located by the AIHN index.

**Figure 10:**
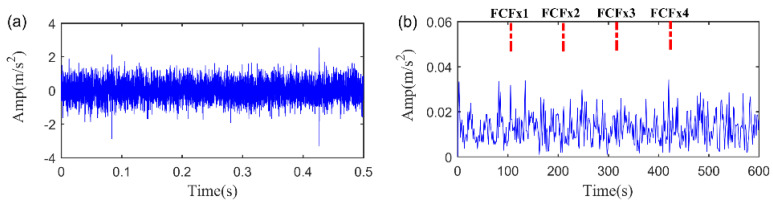
The simulation signal. (**a**) The time waveform. (**b**) The envelope spectrum.

The SNR of the simulation signal is set to −12.80 dB. The time waveform and its frequency spectrum of the simulation signal are depicted in Figure 10. We can find that fault related feature cannot be observed in time domain. Moreover, we cannot find any indications for the fault characteristic frequency (FCF) of the rolling bearing in the envelope spectrum, since that the weak fault feature is totally submerged by the heavy background noise. 

**Figure 11:**
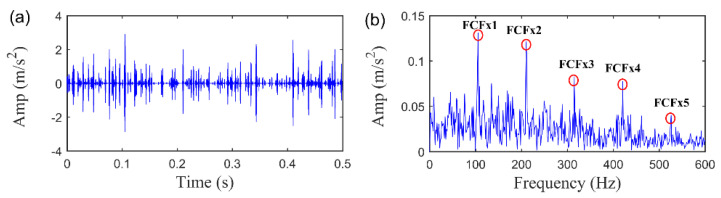
The extracted feature signal using SLRGSD approach. (**a**) The time waveform. (**b**) The envelope spectrum.

Initially, we can obtain the extracted fault feature signal and its envelope spectrum using the proposed SLRGSD algorithm, as displayed in Figure 11. According to the regularization parameter optimization strategy proposed in Section 4.3. The parameters of SLRGSD are set as follows: delay time τ=15,∂=0.5, λ=0.425. From Figure 11, it is evidently that the quasi-periodic impulse fault feature can be identified. Meanwhile, the FCF and its spectral peaks can be accurately identified in the envelope spectrum. Therefore, it is reasonable to assert that the fault happened.

**Figure 12:**
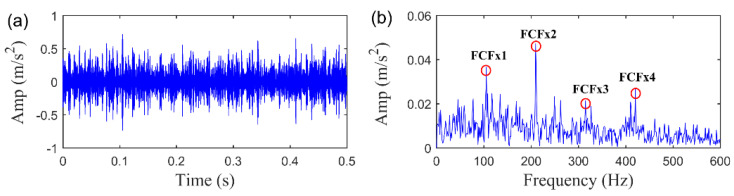
The extracted feature signal using RSVD approach. (**a**) The time waveform. (**b**) The envelope spectrum.

The same simulation signal is processed by the baseline RSVD method. To make a fair comparison, the delay time of RSVD is also set as τ=15. The fault detection results are shown in Figure 12. We can observe that the impulsive fault pattern in the time waveform attained by RSVD is contaminated by the heavy noise although the feature related spectral peaks in the envelope spectrum can be identified. Moreover, the fault related components detected in the envelope spectrum by RSVD is much less clear than SLRGSD approach. Thus, we can assert that the SLRGS property is essential to enhance the fault feature of the rolling bearing for the proposed method.

**Figure 13:**
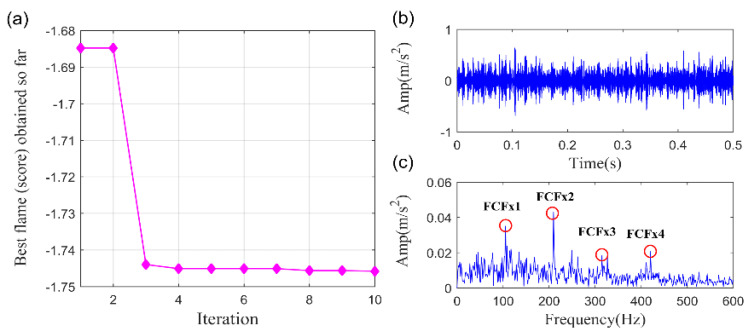
The extracted signal by PO-VMD method. (**a**) The MFO convergence curve. (**b**) The time waveform. (**c**) The envelope spectrum.

Furthermore, the parameters optimized VMD (PO-VMD) [57] method is explored to implement the same task for comparison. Subsequently, we plot the recovered fault feature. The MFO convergence curve for finding the minimum −PI is displayed in Figure 13a while the time waveform and the frequency spectrum of the extracted fault feature are displayed in Figure 13b,c, respectively. The optimized parameters are gained as: α=1658, K=5. It can be found that the FCF and its high-order harmonics could be detected in the envelope spectrum. However, plenty of noise remains in the extracted fault feature and the higher-order FCFs are not clearly enough. As a result, we can assert that the fault feature obtained by PO-VMD method is not accurate enough as compared with SLRGSD approach.

**Figure 14:**
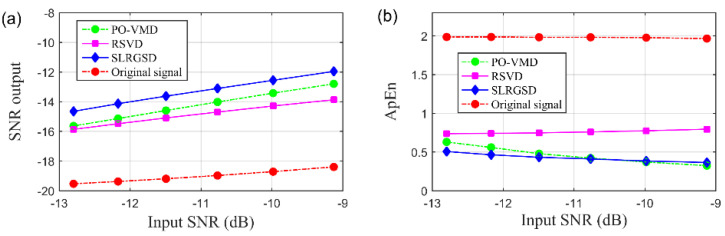
The (**a**) Output SNR and (**b**) ApEn of PO-VMD, RSVD and the proposed SLRGSD methods of processing the simulation signals with different input SNR.

Finally, the ApEn and SNR output indicators introduced in Section 4.1 are employed to perform the quantitative comparison of the SLRGSD, RSVD and PO-VMD method. Without loss of generality, six groups of simulation signals with different input SNR serve as the benchmark signal to achieve this task. The comparison results are demonstrated in Figure 14. Several observations can be concluded: (1) Overall, the proposed SLRGSD has the best performance of extracting the fault feature compared with RSVD and PO-VMD method. The output SNR of the extracted features by SLRGSD is approximately 1.6 dB larger than RSVD while and is about 1.1 dB larger than PO-VMD method. (2) The ApEn of the extracted features obtained by SLRGSD is approximately the smallest. Therefore, the SLRGSD has a distinct advantage over the RSVD and the PO-VMD method. 

## 5. Experimental Verification

In this section, two kinds of experiments are employed to illustrate effectiveness and robustness of the proposed SLRGSD method for bearing fault diagnosis. The first one is the artificial inner and outer race fault experiment of the rolling bearing, which is used to evaluate the performance of proposed method under heavy background noise. The second one is the wind turbine bearing early fault experiment, aiming at assessing the incipient fault detecting ability of the proposed method. In addition, the outperformance of the proposed SLRGSD method over the start-of-the-art methods are demonstrated. 

### 5.1. Inner and Outer Race Fault Diagnosis 

**Figure 15:**
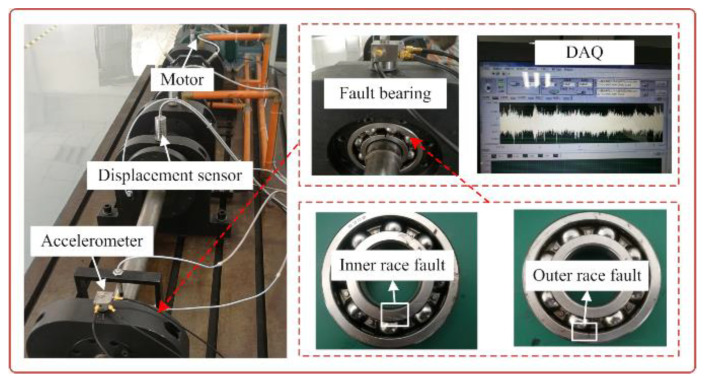
The test rig of artificial bearing fault.

In this experiment, the artificial fault generated by the electric discharge is seeded at the inner race and outer race of the rolling bearing. The shaft hosts one test bearing and three support bearings for the testing rig, as displayed in Figure 15. A HD-YD-232 piezoelectric accelerometer is vertical mounted on the bearing house. The fault vibration signals are collected by a data acquisition system with the sampling frequency of 20,480 Hz. The model type of the fault bearing is SKF6308. Its specific parameters are detailed as follows: pitch diameter D= 77.7 mm, rolling element diameter  d =15.1 mm, contact angle $\theta =0^{o}$, ball number Z=8. The rotating shaft speed is set to 600 rpm (the rotating frequency is equal to $f_{r}=10\;\mathrm{Hz}$). The fault characteristic frequencies of the inner race BPFI and outer race BPFO can be obtained according to the geometry size and the rotating frequency based on the theoretical calculation formula [9,10], which are BPFI = 48.75 Hz and BPFO = 30.63 Hz, respectively. It is worth noting that in order to further demonstrate the effectiveness of proposed method, a little Gaussian white noise is added to the original fault signal. 

#### 5.1.1. Inner Race Fault Diagnosis

**Figure 16:**
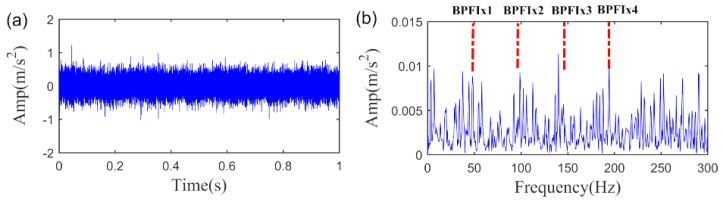
The inner race fault signal added with Gaussian noise. (**a**) The time waveform. (**b**) The envelope spectrum.

We first analyze the vibration signal with inner race fault, where a segment of original signal with 1s is employed to validate the performance of the SLRGSD algorithm. Note that the Gaussian noise with intensity of 17.75 dBm is added into the original signal. The time waveform of the signal and its envelope spectrum are displayed in Figure 16. Apparently, no fault related feature can be observed in the time domain as it is completely contaminated by the heavy noise. Meanwhile, the interference harmonics are dominated in the envelope spectrum. Thus, it is hard to identify that the inner race fault happened. 

**Figure 17:**
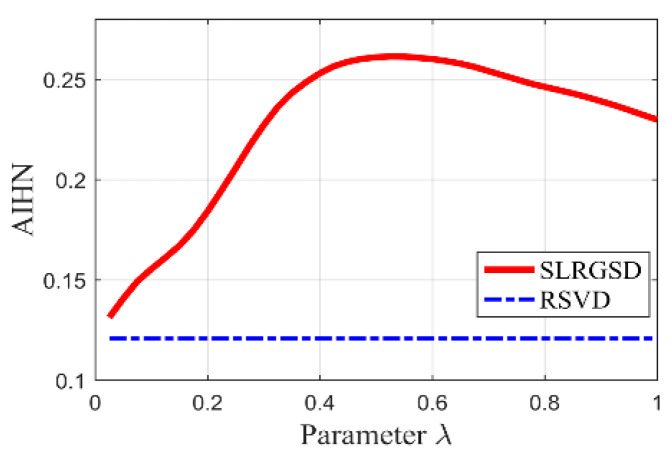
The relationship between the parameter λ and the AIHN value for the inner race fault signal.

**Figure 18:**
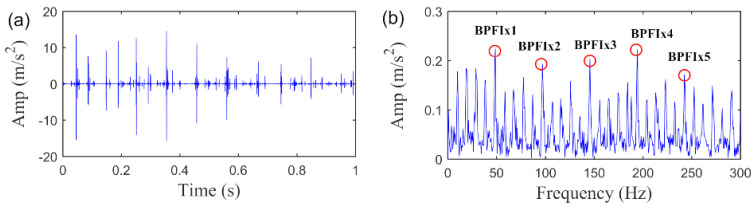
The extracted fault feature signal of inner race fault by SLGRSH method. (**a**) The time waveform. (**b**) The envelope spectrum.

The proposed SLRGSD method is performed to identify the fault feature of the vibration signal. To illustrate the outperformance of the SLRGSD method, the state-of the art RSVD method and the PO-VMD method are also to accomplish the same task. According to the rule proposed in Section 4.3. The parameters are set as: the ∂ is fixed as ∂=0.5 while the delay time τ is fixed as τ=15. Here, using the similar approach proposed in Section 4.4, the AIHN of the extracted feature is utilized to illustrate the optimization of parameter  λ. The interval is set 0.025. The trajectory of AIHN of the extracted fault feature is displayed in Figure 17. Interestingly, we can observe that the trajectory of AIHN value in Figure 17 has good agreement with Figure 9. That is, in the beginning, AIHN value gradually increases with the growing of parameter λ. Nevertheless, after the AIHN value reaches the maximum, the performance degradation of SLRGSD method appears with the further increasing of the parameter λ. One possible reason is that the low rank property is suppressed while the group sparse property playing a leading role in the regularization model of SLRGSD approach. After the short analysis, it is not hard for us to find that the maximum AIHN equals to 0.2614 when the parameter λ=0.525. In this case, we observe the recovered fault feature using the SLRGSD method, as shown in Figure 18. In Figure 18a, it can be found that the noise is significant eliminated in the time domain. Moreover, the fault characteristic frequency BPFI and its harmonics are accurately captured, as shown in Figure 18b. Therefore, we can make a clear conclusion that the fault happened in the inner race. 

**Figure 19:**
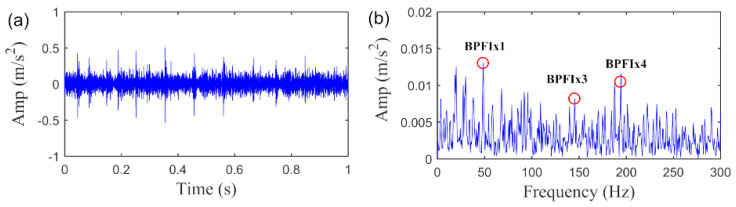
The extracted fault feature signal of inner race fault by RSVD method. (**a**) The time waveform. (**b**) The envelope spectrum.

To further evaluate the role of SLRGS property and verify the superiority of the SLRGSD method, we compare it with the baseline RSVD method. The delay time τ of RSVD method is set as τ=15. Leveraging the AIHN index, we can investigate the informative fault feature extracted by the RSVD method. In this regard, we can find the AIHN value of the fault feature obtained by RSVD is 0.1208, which is less than half of the value obtained by SLRGSD approach, as shown in Figure 16. In addition, Figure 19 plots the time waveform and the envelope spectrum of the detected fault feature pattern. Broadly speaking, the RSVD can also identify the fault feature because the fault characteristic frequency BPFI can be observed in the envelope spectrum. Nevertheless, plenty of noise is still preserved in the fault feature. Clearly, the above evidences fully demonstrate that the SLRGSD can obtain much better result than the RSVD method. It can be affirmed that the SLRGS property is key building blocks for enhancing the fault feature for bearing fault diagnosis, which is the main motivation and contribution of this research.

**Figure 20:**
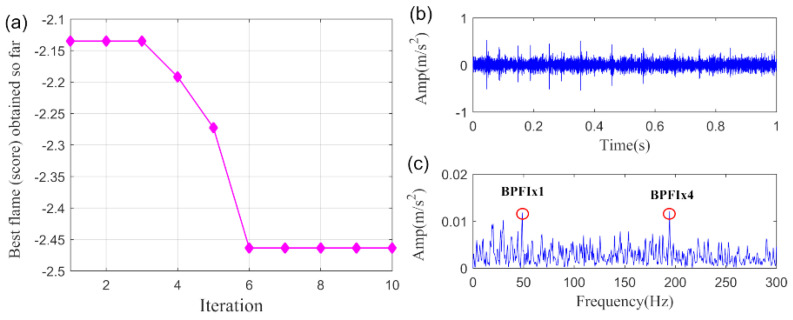
The extracted signal of inner race fault by PO-VMD method. (**a**) The MFO convergence curve. (**b**) The time waveform. (**c**) tThe envelope spectrum.

To demonstrate the superiority of proposed SLRGSD method, the PO-VMD method is employed to process same vibration signal. According to the parameter optimization strategy, the MFO convergence curve for finding the minimum −PI is shown in Figure 20a. The optimized mode frequency bandwidth control parameter ∂ and the number of modes K are gained as α=1000,K=2. The extracted fault feature and its envelope spectrum are presented in Figure 20b,c, respectively. It can be found that only the fault characteristic frequency BPFI and its fourth high-order harmonic can be detected. There are still lots of noise in the extracted fault feature. The results indicate that the fault feature obtained by PO-VMD is less clear than the proposed SLRGSD method.

#### 5.1.2. Outer Race Fault Diagnosis

**Figure 21:**
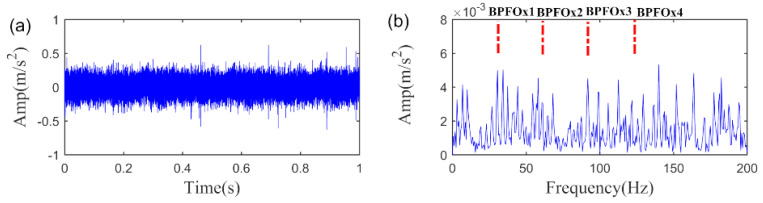
The outer race fault signal added with Gaussian noise. (**a**) The time waveform. (**b**) The envelope spectrum.

To further prove the effectiveness of the proposed SLRGSD algorithm, the outer race fault vibration signal is also employed for analysis. Similarly, the Gaussian noise with intensity of 11.73 dBm is added into the original signal. The time waveform and the envelope spectrum of the signal are shown in Figure 21. As can be observed from Figure 21, the background is too heavy so that weak fault feature is too hard to be detected. More importantly, the fault frequency spectral peaks BPFO in the envelope spectrum cannot be located. It is intensely difficult for us to assert that outer race fault happened.

**Figure 22:**
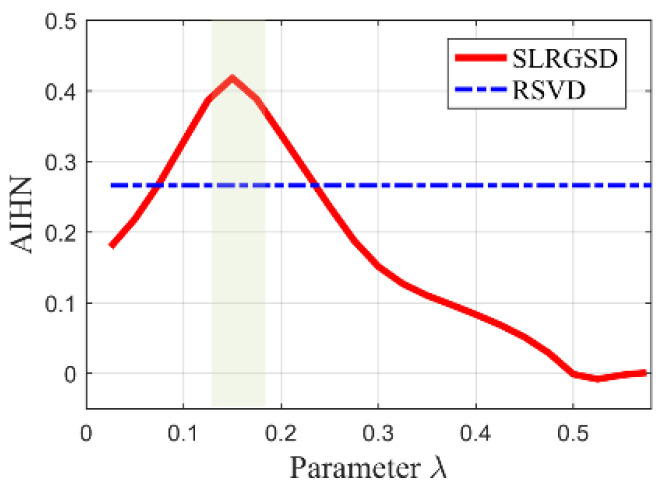
The relationship between the parameter λ and the AIHN value for the outer race fault signal.

Therefore, the proposed SLRGSD method is taken to identify the fault feature. Similarly, parameters m and ∂ are set as m=15,∂=0.5. We also search the maximum parameter λ to illustrate the SLRGS property. The step size is set to 0.025. The AIHN trajectory of the fault feature obtained by SLRGSD is presented in Figure 22. It is worth noting that there are only 23 points on the tracks since the AIHN value is less than zero after the 24th calculation. We make a detailed analysis for Figure 22. Apparently, the changing trend of the AIHN value obeys the same rule. When the parameter λ is small, the low rank property is a leading role. Then the weight of group sparsity is increased when the parameter λ grows. When the parameter λ reaches the optimal point, the combination of low rank and group sparsity works the best. It can be observed that when the optimized parameter λ=0.15, the AIHN has the maximum value 0.4183. 

**Figure 23:**
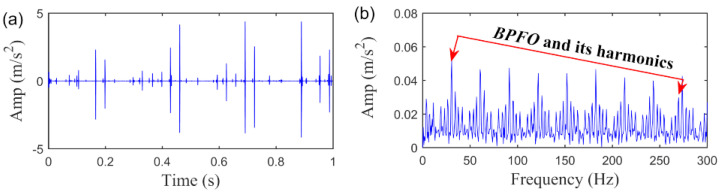
The extracted fault feature signal of outer race fault by SLGRSD method. (**a**) The time waveform. (**b**) The envelope spectrum.

**Figure 24:**
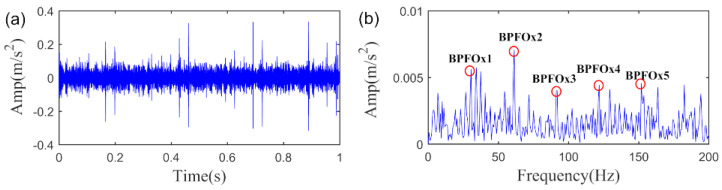
The extracted fault feature of outer race fault signal by RSVD method. (**a**) The time waveform. (**b**) The envelope spectrum.

We plot the fault feature obtained by SLRGSD method based on the above parameters. The results are shown in Figure 23. Obviously, the periodic fault feature is remarkably enhanced in time domain. Moreover, it is evidently that there is outer race fault in the rolling bearing as the fault feature frequency BPFO and its high-order harmonics are quite distinct. On the other hand, as can be seen in Figure 21, the AIHN value of the fault feature detected by RSVD is only 0.266, which is far less than the AIHN value attained by SLRGSD. Furthermore, the fault feature pattern obtained by RSVD method is displayed in Figure 24. It can be found that the residual noise is still very heavily in the time domain and envelope spectrum. The fault feature frequency BPFO and its harmonics cannot be reliably identified. The outperformance of the SLRGSD approach is confirmed. The above analysis proves that the SLRGS property is the key step for improving the performance of the SLRGSD method.

**Figure 25:**
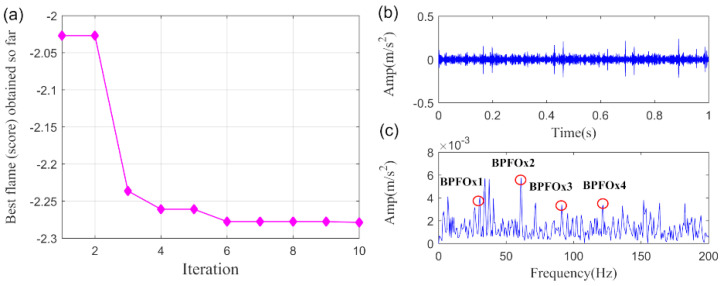
The extracted signal of outer race fault by PO-VMD method. (**a**) The MFO convergence curve. (**b**) The time waveform. (**c**) The envelope spectrum.

In a similar way, the PO-VMD method is applied to analyze the vibration signal with outer race fault for comparison. The MFO convergence curve of searching the minimum −PI is shown in Figure 25a. The maximum PI value is 2.279. The optimized parameters satisfy α=4256, K=7. The extracted fault feature and its envelope spectrum are shown in Figure 25b,c, respectively. We can observe that the faut frequency BPFO can be detected in the envelope spectrum by the PO-VMD method. However, there are many interference harmonics in the extracted signal by PO-VMD. As a result, the fault feature obtained by PO-VMD is indistinct if we compared it with results obtained by SLRGSD method.

### 5.2. Application to Wind Turbine Bearing Fault Diagnosis

**Figure 26:**
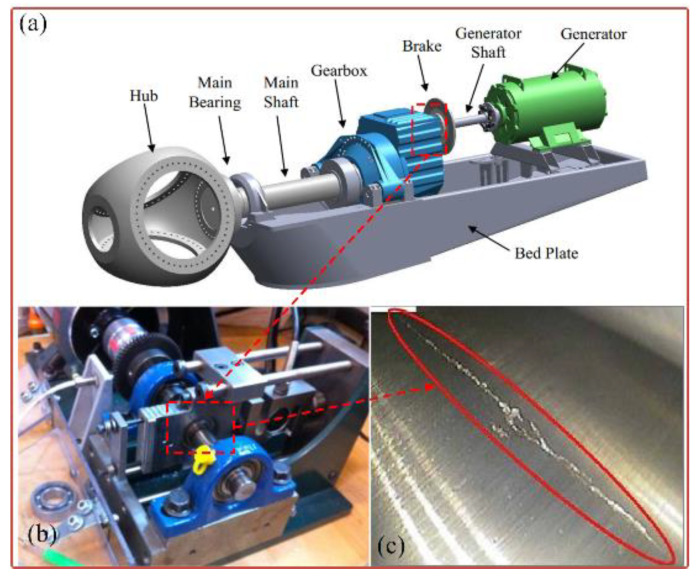
(**a**) The schematic diagram of the wind turbine (**b**) The test rig of wind turbine bearing fault. (**c**) The observed wind turbine bearing inner race fault.

**Figure 27:**
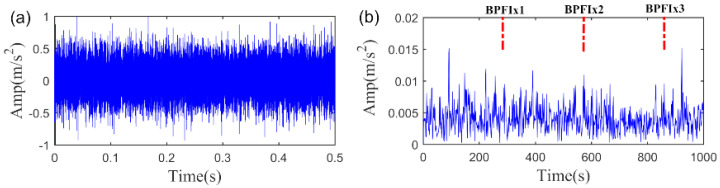
The early wind turbine bearing fault signal. (**a**) The time waveform. (**b**) The envelope spectrum.

In this section, the bearing fault signal of the wind turbine is employed to verify the effectiveness of the proposed SLRGSD method. The test rig is shown in Figure 26. The data acquisition system is designed by the Green Power Monitoring Systems in the USA [58]. In the testing process, the MEMS accelerometer (Model: ADXL001) was radially installed on the gearbox bearing support, as shown in Figure 26a. The sample frequency was set to 97,656 Hz. Each sample was collected by 6s. The records of 50 days were considered for the original signal. At the end of the experiment, an inner race fault was observed on the tested bearing (Model: 32222-J2-SKF). The specification parameters of the bearing are shortly introduced as follows: the outer diameter of the bearing is 200 mm while the bore is 110 mm. There are 20 rolling elements at a 16° taper angle of the bearings. The running speed of the shaft is 1800 rpm (the rotating frequency is equal to $f_{r}=30\;\mathrm{Hz}$). Based on the geometry parameter and the running speed of the rolling bearing, we can obtain its fault characteristic fault frequency, which is BPFI = 286 Hz. According to Ref. [58], the rolling bearing is in the initial degenerate state for the 10th day. Therefore, we can assume that the fault signal in the 11th day is the incipient fault feature signal. To this end, the fault signal in the 11th day is performed to validate the effectiveness of the proposed SLRGSD algorithm. The fault signal in time domain and the envelope spectrum domain are presented in Figure 27. Apparently, the early fault feature is totally submerged by the heavy background noise and no fault-related information can be detected. 

**Figure 28:**
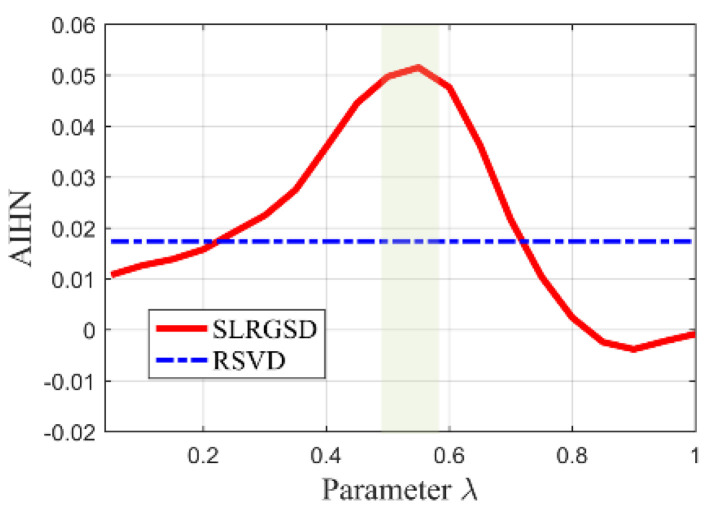
The relationship between the parameter λ and the AIHN value for the wind turbine fault signal.

**Figure 29:**
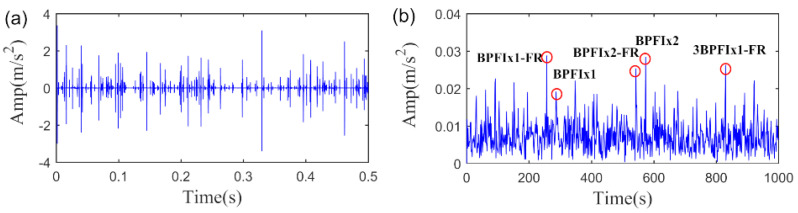
The extracted fault feature signal of early wind turbine bearing fault by SLGRSH method. (**a**) The time waveform. (**b**) The envelope spectrum.

Firstly, the signal described in Figure 27 is processed by the SLRGSD method. Note that the original signal is normalized for further processing. Like the previous analysis, the parameter m and α are also set as m=15,∂=0.5. Here, the AIHN is utilized to indicate the changing trend for selecting the appropriate parameter λ. Note that the parameter λ increases from 0 to 1 with an interval of 0.025. The trajectory of AIHN is shown in Figure 28. One can see that the trajectories of AIHN follow a similar pattern, that is, the AIHN value achieves the maximum with the suitable combination of the low rank and group sparse property. Besides, the maximum AIHN is 0.05153 when the optimized parameter λ=0.55. In this scenario, the time waveform and the envelope spectrum of the detected fault feature are displayed in Figure 29. It can be found that the fault feature frequency BPFI, its spectral peaks 2BPFI, 3BPFI and the side frequency BPFI-FR, 2BPFI-FR, 3BPFI-FR can be clearly identified. Therefore, the incipient inner race fault of the rolling bearing can be confirmed.

**Figure 30:**
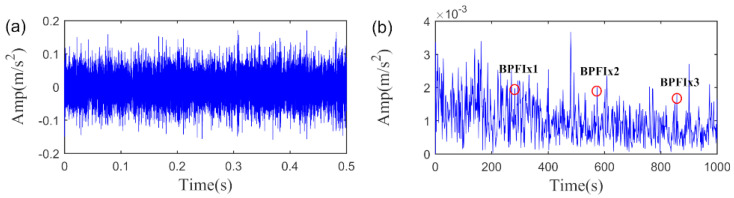
The extracted fault feature signal of early wind turbine bearing fault by RSVD method. (**a**) The time waveform. (**b**) The envelope spectrum.

For comparison, the RSVD method is employed to fulfil the same fault detection task. We first calculated the AIHN value of the extracted fault feature using the RSVD method. Notably, the AIHN equals to 0.0174, which is roughly one-third of the maximum AIHN value obtained by SLRGSD method. In addition, as shown in Figure 30, it is hard to affirm that the early inner race fault happened since the noise is still very strong in the extracted fault feature obtained by RSVD method. The comparison results verify the obvious advantages of the proposed SLRGSD method over the baseline RSVD method. Therefore, it can be confirmed again the SLRGS property is a solid foundation for boosting the performance of SLRGSD method for the rolling bearing fault diagnosis.

**Figure 31:**
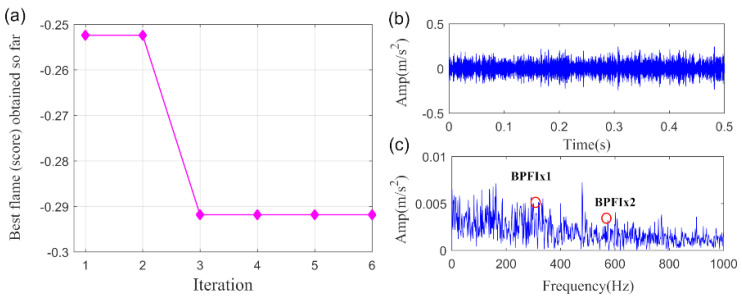
The extracted signal of early wind turbine bearing fault by PO-VMD method. (**a**) The MFO convergence curve. (**b**) The time waveform. (**c**) The envelope spectrum.

Lastly, the PO-VMD method is adopted to process the bearing fault signal of the wind turbine to verify the superiority of the proposed method. The MFO convergence curve for finding the minimum-PI is shown in Figure 31a. The obtained optimized parameters are α=3263, K=5. The extracted fault feature and its envelope spectrum are displayed in Figure 31b,c, respectively. Obviously, it is hard for us to confirm that the inner race fault happened according to the extracted fault feature through the PO-VMD method. In summary, the comparison results highlight the superiority of the proposed SLRGSD method.

## 6. Conclusions

To address the defect of the current Hankel matrix based SVD method for bearing fault diagnosis, a novel SLRGSD method is proposed in this research. The highlight of the proposed SLRGSD approach is that the SLRGS property of fault feature is first revealed for enhancing the fault diagnosis performance. Specifically, the proposed framework combines the weighted nuclear norm and the groups sparse to promote the SLRGS property for bearing fault diagnosis. The incremental-proximal descend algorithm is further utilized to obtain the closed solution for the optimization problem. The PI index evaluation and the configuration of the main regularization parameters are discussed in the numerical analysis. Moreover, leveraging the AIHN indicator, the role of the SLRGS property is profoundly demonstrated in the numerical simulation. The results validate that the SLRGS property is a key cornerstone to enhance the performance of SLRGSD method, which is the main motivation and contribution of this research. The proposed SLRGSD method has been successfully applied to process the vibration signals generated by the bearing fault test rig and the wind turbine bearing fault platform. The superiority over the state-of-the-art methods such as RSVD and PO-VMD is verified, indicating that the proposed SLRGSD method can be a protentional tool to enhance the incipient fault feature for bearing fault diagnosis.
